# The Impact of Coronavirus Disease 2019 on the Disease Pattern of Oral and Maxillofacial Surgery Inpatients: A Comparative Study

**DOI:** 10.3389/fmed.2021.613663

**Published:** 2021-04-29

**Authors:** Jingya Jane Pu, Colman Patrick McGrath, Yiu Yan Leung, Wing Shan Choi, Wei-fa Yang, Kar Yan Li, Yu-xiong Su

**Affiliations:** ^1^Division of Oral and Maxillofacial Surgery, Faculty of Dentistry, The University of Hong Kong, Pokfulam, Hong Kong; ^2^Division of Applied Oral Sciences and Community Dental Care, Faculty of Dentistry, The University of Hong Kong, Pokfulam, Hong Kong

**Keywords:** COVID-19, coronavirus, oral and maxillofacial surgery, oral cancer, essential services, healthcare, pandemic, disease pattern

## Abstract

**Objective:** Oral and maxillofacial surgery (OMFS) is a high-risk specialty involving airway and aerosol-generating procedures, which is potentially of more risk in the era of coronavirus disease 2019 (COVID-19). We aimed to identify the impact of COVID-19 on the disease pattern of OMFS inpatients and surgeries under general anesthesia in a comparative study.

**Materials and Methods:** We reviewed the admission and operating theater records of OMFS patients from Jan 1 to Aug 31 in 2020 and 2019. The total number of cases, presenting disease patterns, and proportion of essential and non-essential medical services were compared between 2020 and 2019.

**Results:** There were 664 admissions and 356 general anesthesia surgical procedures included in this study. Both admission and surgery numbers were significantly reduced in 2020, compared with 2019 (*p* = 0.012 and 0.007, respectively). The proportion of malignancy cases increased significantly, whereas that of cleft lip and palate and temporomandibular disorder (TMD) decreased. There was a significant increase in the proportion of essential services compared with non-essential services in 2020 compared with 2019.

**Conclusion:** Our results first reported the epidemiological data of the impact of COVID-19 on OMFS disease pattern in a comparative study. The change of disease pattern and caseload will have a long-term impact on OMFS patient care, education, and training during the pandemic. Our paper provides evidence for health policy makers to consider the relocation of medical resources and optimization of medical education and services.

## Introduction

The pandemic outbreak of severe acute respiratory syndrome coronavirus-2 (SARS-CoV-2), which is also known as coronavirus disease 2019 (COVID-19), has been posing major impacts on health care (1). Since the declaration of the World Health Organization (WHO) of COVID-19 outbreak as an international public health emergency, different health-care policies have been adopted by countries worldwide. In general, there is a reduction in the number of elective procedures due to the risk of COVID infection and limited medical resources (2).

During the pandemic, prioritizing medical services is crucial to avoid collapse of medical systems. The health-care system failures during the outbreak of Ebola virus in 2014–2015 contributed to the increased number of deaths caused by measles, malaria, HIV/AIDS, and tuberculosis (3). Therefore, maintaining the essential medical services during an outbreak is of premier importance to avoid increased morbidity and mortality contributed indirectly by the insufficiency or inappropriate distribution of medical resources. According to the guidance by the WHO, priorities of elective surgeries change over time and vary from country to country. The main factors to consider while scheduling the elective treatments including the level of outbreak, availability of the health-care service in the area, and estimated length of the shortage of services (4).

**Figure 1:**
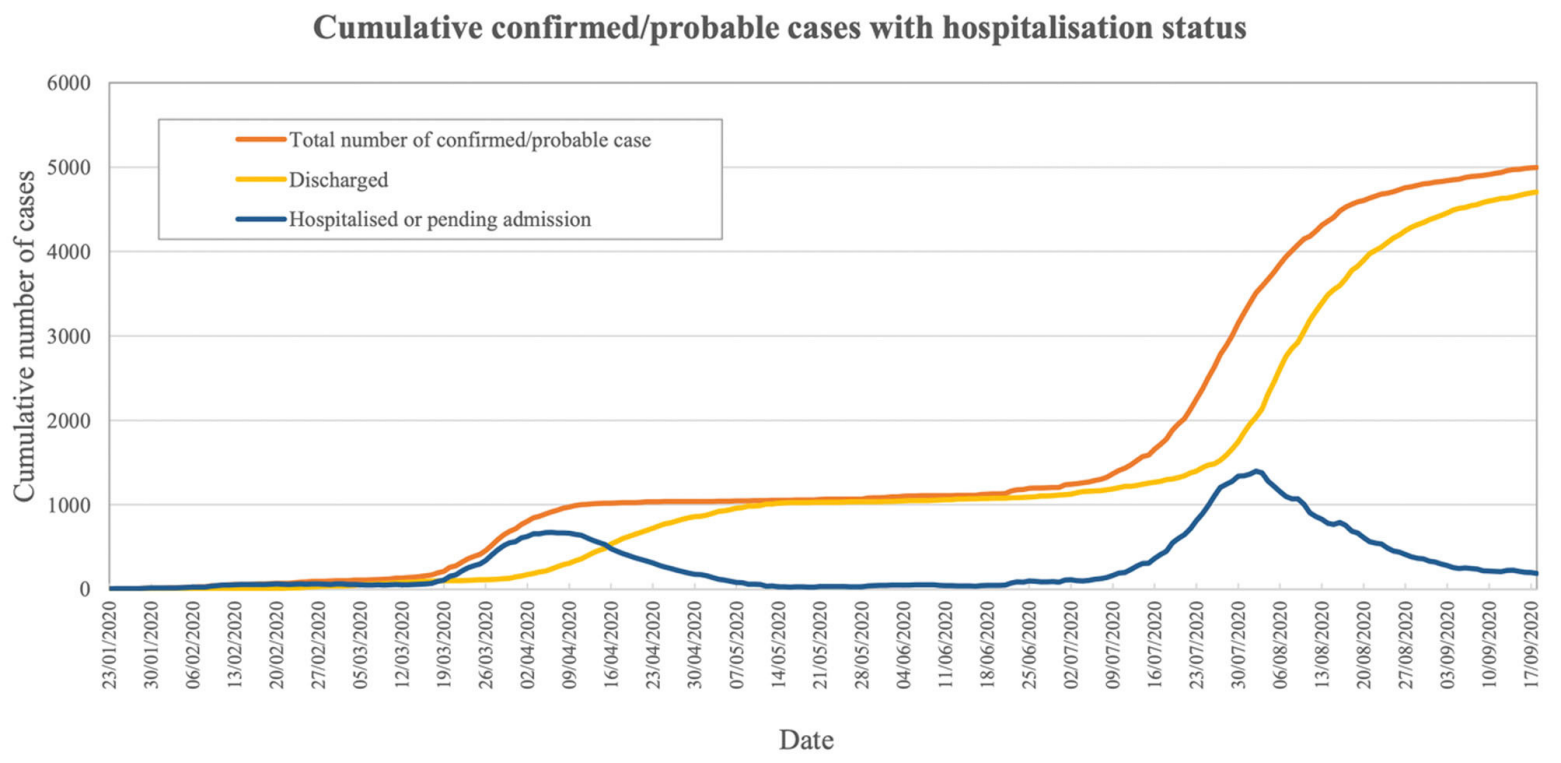
Cumulative confirmed/probable cases with hospitalization status (5).

Despite the high population mobility and density in Hong Kong, the citizens, government, and medical professionals have been striving for a reasonable control of the pandemic. From January 1, 2020, to September 19, 2020, there were a total of 5,010 cases of COVID-19 documented by the Department of Health of Hong Kong. Most of the cases were discharged after full recovery (4,708/5,010), while 103 (2.1%) were fatal. The number of patients hospitalized or pending admission was kept below 1,000 most of the time (Figure 1), which was within the isolation ward capacity in Hong Kong (5).

Oral and maxillofacial surgery (OMFS) is considered as one of the high-risk specialties due to the involvement of airway and aerosol-generating procedures. However, the impact of COVID-19 on the disease pattern of inpatients and operations in OMFS has yet to be studied. Globally, OMFS services were severely affected during the pandemic, while we have been keeping a certain level of OMFS services including elective cases in Hong Kong. This provides an ideal model to study the service pattern changes of OMFS during the COVID-19 era. Although WHO suggested health conditions and acute presentations that require time-sensitive intervention to be considered as essential medical services, the context-relevant definition in OMFS field is still lacking (4). The aim of this study is to investigate the impact of COVID-19 on the disease pattern in OMFS inpatients and surgeries under general anesthesia and to identify the context-relevant essential services in OMFS field for future reference when it comes to policy making and resource distribution.

## Materials and Methods

The study was approved by the Institutional Review Board of the University of Hong Kong (IRB Reference Number: UW 20-684), with the patient informed consent waived. We performed a comparative study in the Department of Oral and Maxillofacial Surgery, University of Hong Kong, Queen Mary Hospital, a tertiary hospital in Hong Kong. The study consisted of two parts. The first part focused on the distribution of inpatients admitted to the OMFS ward, Queen Mary Hospital; while the second part analyzed the cases operated in the operating theater (OT) under general anesthesia by the OMFS team. Records from January 1 to August 31 in 2019 and 2020 were reviewed and divided into eight groups below, with dental extraction cases excluded.

1. Dentofacial deformities
2. Benign pathologies
3. Malignancy
4. Dentoalveolar diseases
5. Infections
6. Cleft lip and palate
7. Temporomandibular joint diseases
8. Others (sleep endoscopy, trauma, nerve repair, etc.).

The total number of cases and proportions of different categories of cases were documented and compared so as to illustrate the difference in disease pattern between 2019 and 2020. Cases of malignancy, infection, and trauma were considered as in need of time-sensitive management, thus proposed as “essential medical services” according to the WHO guideline (4).

Polymerase chain reaction screening test for SARS-CoV-2 infection was conducted within 24 h before the operation or admission. Patients would be operated only if they had no travel history within 14 days, had not been in close contact with confirmed cases, were showing no symptom suggesting possible COVID-19 infection, and had negative PCR results. Patients in close contact with confirmed cases were observed for at least 14 days before presenting to the hospital unless they require a life-saving emergency surgery.

All statistical analyses were performed using IBM SPSS Statistics Version 26. Binomial test was performed to compare the difference in total number of cases, while chi square test was used to compare the proportions of essential medical services as well as different groups of cases. Bonferroni adjustment was applied for multiple group comparisons. All tests and reported *p*-values were two-sided. A *p* < 0.05 was considered statistically significant.

## Results

### Comparison of Disease Distribution of Inpatients

**Figure 2:**
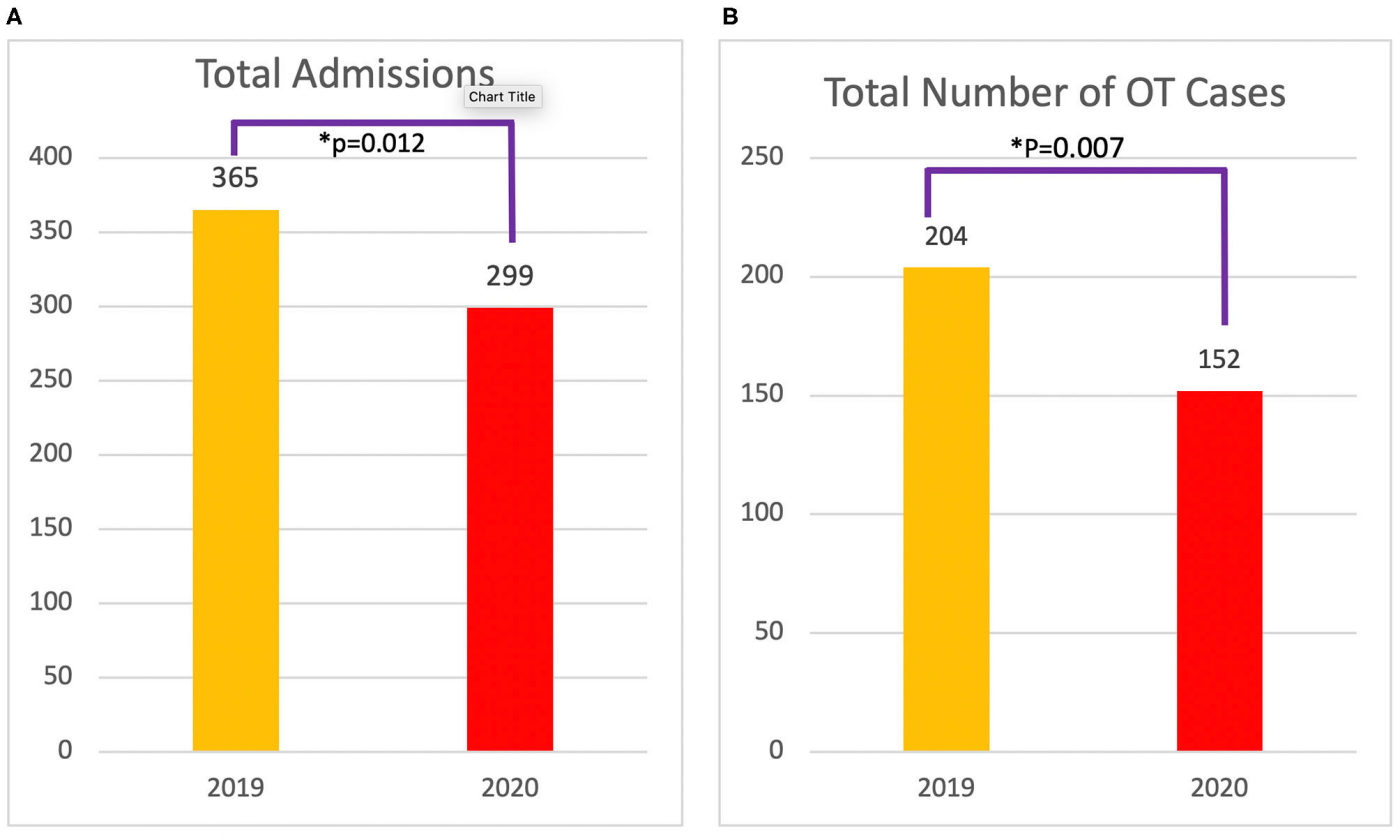
Total number of admissions **(A)** and operating theater (OT) operations **(B)** from Jan 1 to Aug 31 in 2019 and 2020.

A total of 664 patients were admitted in the designated periods. Significantly fewer patients were admitted to the hospital in 2020 compared with 2019 (*p* = 0.012) (Figure 2). None of the patients tested positive for COVID-19 upon admission or developed COVID-19 infection during their inpatient stay. No health-care professional in our team developed symptoms or tested positive for COVID-19 during the studied period.

**Figure 3:**
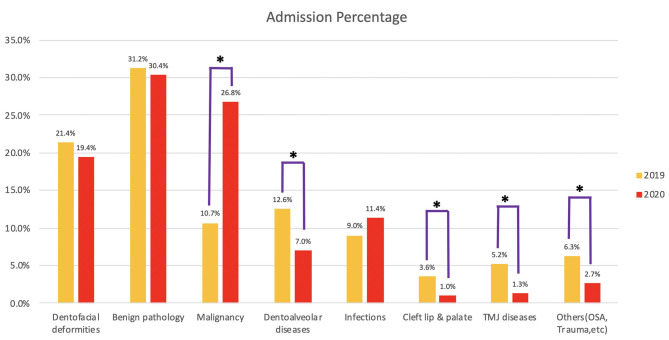
Percentage of different categories of admissions. *Groups with significant difference between 2019 and 2020 after Bonferroni adjustment.

**Figure 4:**
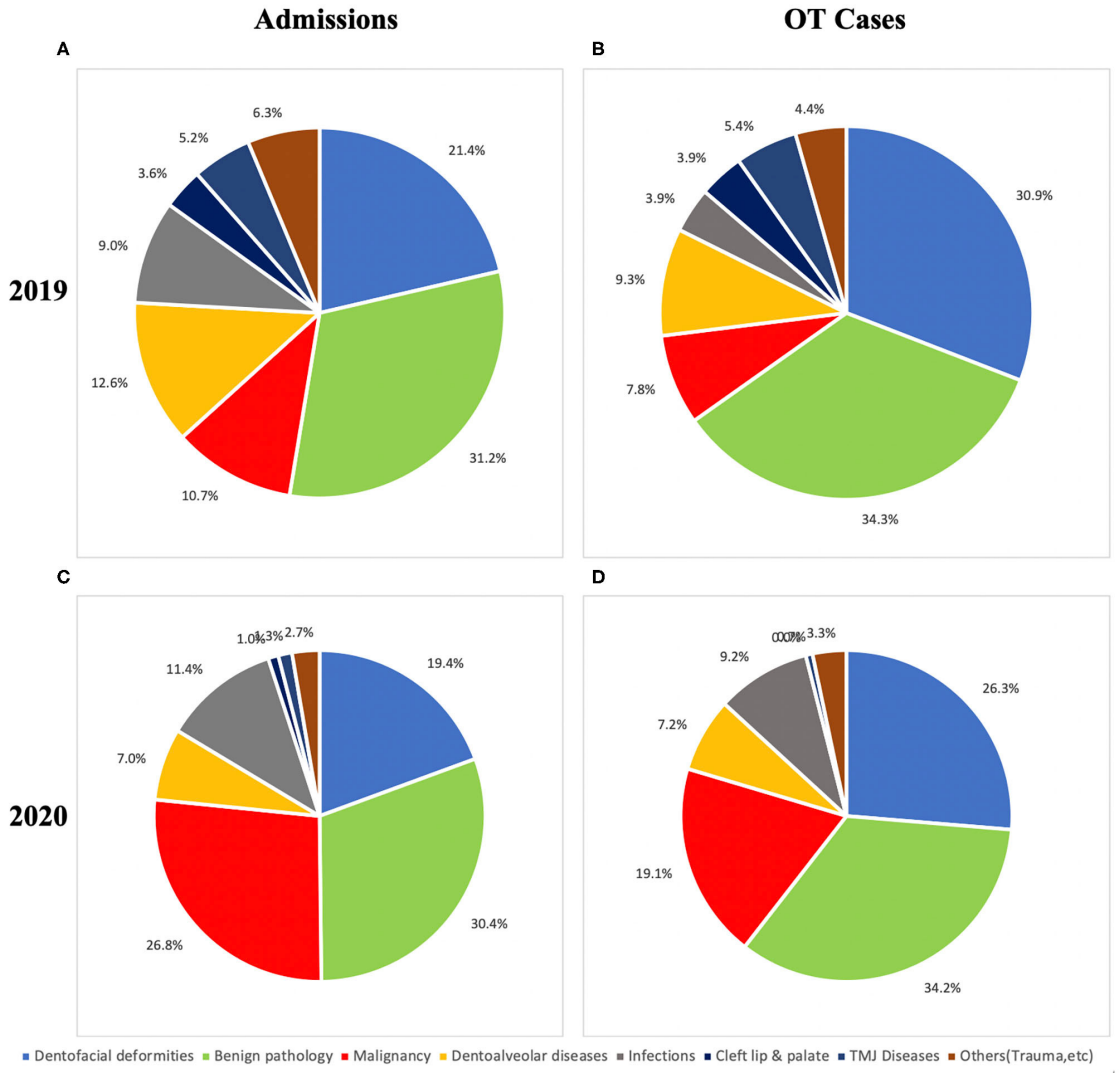
Distribution of admissions and operating theater (OT) cases. **(A)** Admissions in 2019. **(B)** OT cases in 2019. **(C)** Admissions in 2020. **(D)** OT cases in 2020.

The numbers and percentages of different groups of admissions are shown in Table 1 and Figure 3. A significantly larger proportion of patients with malignancies were admitted in 2020 compared with 2019. In contrast, significantly fewer patients were admitted for dentoalveolar diseases, cleft lip and palate, and temporomandibular joint diseases. The admissions for the category of “others” also showed significant difference probably due to the decreased number of admissions for obstructive sleep apnea patients in 2020. The proportion of patients admitted for dentofacial deformity, benign pathology, and infections were similar in 2019 and 2020. Percentage distributions of different groups of admissions in 2019 and 2020 are shown in Figure 4.

**Table 1:** Number of admissions and OT cases in from Jan 1 to Aug 31 in 2019 and 2020.

	**Admissions**					**OT**				
	**2019**		**2020**		***p*-value**	**2019**		**2020**		***p*-value**
Dentofacial deformities	78	21.4%	58	19.4%	<0.001	63	30.9%	40	26.3%	0.001
Benign pathology	114	31.2%	91	30.4%		70	34.3%	52	34.2%	
Malignancy	39	10.7%	80	26.8%		16	7.8%	29	19.1%	
Dentoalveolar diseases	46	12.6%	21	7.0%		19	9.3%	11	7.2%	
Infections	33	9.0%	34	11.4%		8	3.9%	14	9.2%	
Cleft	13	3.6%	3	1.0%		8	3.9%	0	0.0%	
TMJ diseases	19	5.2%	4	1.3%		11	5.4%	1	0.7%	
Others (OSA, trauma, etc.)	23	6.3%	8	2.7%		9	4.4%	5	3.3%	
Total	365	100.0%	299	100.0%	0.012	204	100.0%	152	100.0%	0.007

*NS, not significant; OT, operating theater; TMJ, temporomandibular joint; OSA, obstructive sleep apnea*.

### Comparison of Surgery Under General Anesthesia

A total of 356 patients were operated in the theater under general anesthesia. Compared with that in 2019, there was a decrease in total number of cases (*p* = 0.007) (Figure 2). None of the patients operated tested positive for COVID-19.

**Figure 5:**
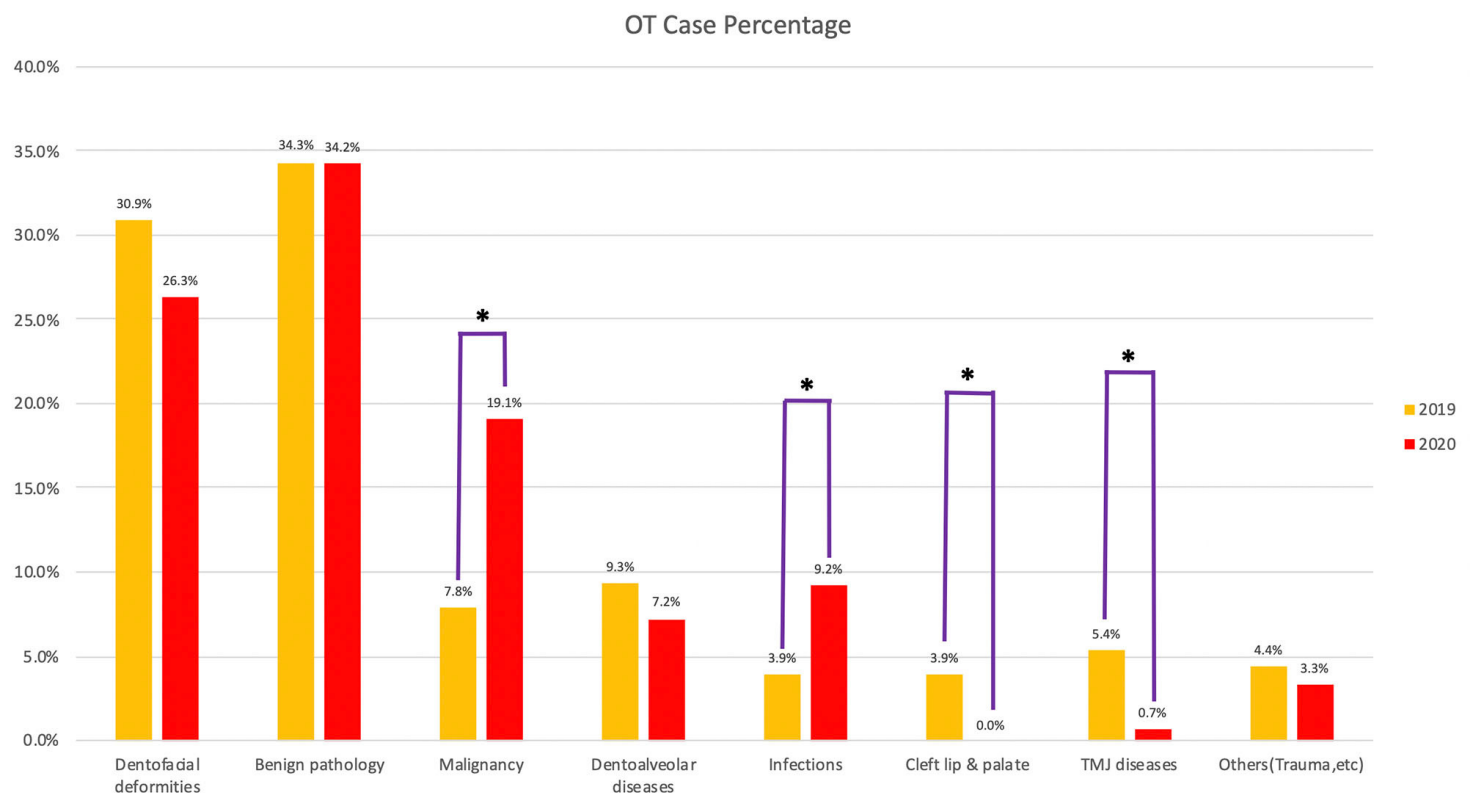
Percentage of different categories of operating theater (OT) cases. *Groups with significant difference between 2019 and 2020 after Bonferroni adjustment.

The number of percentage of cases in different categories is shown in Table 1 and Figure 5. Significant increases in the percentage of malignancy and infection cases were observed in 2020. Priorities were given to the malignant cases with no limitation imposed. Different than most other hospitals during pandemic, we performed microvascular free flap reconstruction for oral malignancy cases as usual. No cleft lip or palate repair surgery was performed in 2020. The difference was significant between 2019 and 2020. Significantly less operations for temporomandibular joint dysfunction were performed in 2020. The proportions of surgeries for dentofacial deformity, benign pathology, dentoalveolar diseases, and others (trauma, nerve repair, etc.) were kept at a similar level. The distribution of cases in 2019 and 2020 is shown in Figure 4.

### Essential Medical Services

**Figure 6:**
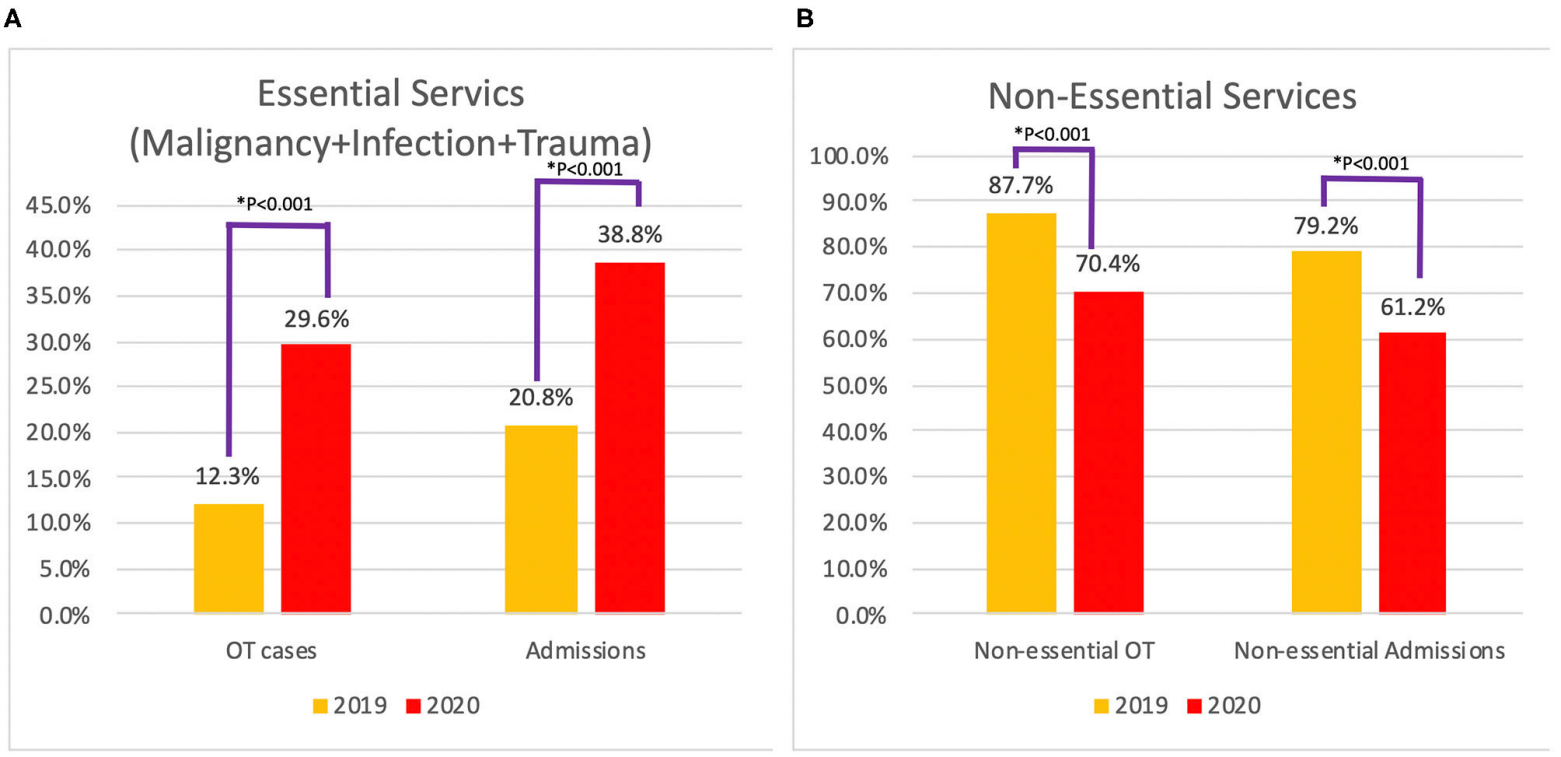
Proportion of essential medical services **(A)** and non-essential services **(B)**.

When malignancy, infection, and trauma were considered as time-sensitive essential medical services, the proportion of essential services increased significantly in both inpatient admissions and surgeries, with the *p* < 0.001. The decrease in the proportion of non-essential cases was also significant in both groups (Figure 6).

## Discussion

Since the outbreak of COVID-19, there have been numerous guidelines on the management of patients during the pandemic (6–8). However, to the best of our knowledge, the current study is the first comparative study to objectively report the significant decrease in total number of patients, the increase in malignancy cases, and essential medical services in OMFS under the influence of COVID-19 pandemic.

Compared with that pre-COVID-19, the total number of cases operated in the OT decreased mainly due to the limited supply of personal protection equipment and the reduction in OT sessions assigned to OMFS. The reduction in the number of admissions could be attributed to reduction in operations as well as decrease of elective inpatient and outpatient services during the outbreak of COVID-19.

The proportion of essential medical services, especially the percentage of malignancy cases, increased significantly under the influence of COVID-19. Although there were no supportive scientific data available so far, various subjective recommendations have been proposed to determine the priority of surgical treatments. From the country with the earliest outbreak, Yang et al. from China defined four categories of OMFS patients according to severity. The critically ill patients include those who require emergency interventions due to life-threatening conditions such as hemorrhage and obstruction of the upper respiratory tract following trauma, tumors, and infections. Subacute patients include those with stable vital signs but requiring urgent interventions such as closed fractures. This is followed by the patients who require expedited interventions including patients diagnosed with malignant tumors, chronic infections, osteomyelitis, etc., while the last group of patients are those who require elective procedures such as those with cleft lip and palate, dentofacial deformities, and benign tumors (9). The malignancy, infection, and trauma cases we included in the “essential medical service” belong to the first three categories in this classification, while the “non-essential medical services” in our study agreed with the last category of elective procedures. Others suggested balancing the risk of delaying the surgery with the resources required for the treatment when determining the priority of surgeries (10, 11). In most situations, priority was given to the essential services when resources were limited. However, when the current pandemic is expected to persist for a relatively long period of time, the changed disease pattern in OMFS might become the new norm. This will have long-term effects in patient care as well as education and training of surgeons. Our data provide reference for policy making and resource distribution in the future.

The precautions we adopted for managing patients in COVID-19 era were similar as the joint recommendations by OMFS in Spain (12). PCR screening test for SARS-CoV-2 infection was conducted within 24 h before the operation or admission. Patients would be operated on only if they had no travel history within 14 days, had not been in close contact with confirmed cases, were showing no symptoms suggesting possible COVID-19 infection, and had negative PCR results. Patients in close contact with confirmed cases were observed for at least 14 days before presenting to the hospital unless they require a life-saving emergency surgery. All cases documented in the current study showed negative results for COVID-19 tests. The operations were performed in the designated COVID-19-negative theater for the OMFS Department in Queen Mary Hospital. WHO guidelines were followed in terms of level of personal protective equipment (PPE) in ward and OT (13, 14). Regardless of the negative results, oral and maxillofacial surgeons were required to wear surgical masks at all time. Aerosol-generating procedures in ward such as changing or removing of tracheostomy tubes and irrigation of intra-oral wound were performed with whole body protection including surgical mask with facial shield, gown, gloves, surgical cap, and eye protection. The same recommendation of protection was applied in the OT. During the period studied, all of our patients and health-care professionals were kept safe from COVID-19 infection, which proved the effectiveness of the precautions we adopted.

In Hong Kong, although the proportion of non-essential services significantly decreased in 2020, we were able to maintain a certain level of service on orthognathic surgery, benign pathology, and dentoalveolar surgeries during the pandemic. This contrasts with the report by Barca et al. about the practice in Italy where all cases treated from Feb 29 to April 16, 2020, were due to either malignancy or trauma (15). A similar situation was observed in the UK where all non-urgent elective surgeries were suspended for 3 months starting April 15, 2020 (16). In the worldwide survey of OMFS surgeons conducted by Maffia et al., only 5.8% of surgeons were still performing orthognathic surgeries (17). This could be attributed to the different levels of pandemic in different parts of the world. In the future, when the pandemic is reasonably controlled, non-essential medical services need to be gradually resumed. The accumulated load of elective procedures might pose a challenge in the recovery and post-recovery periods. Our experience proved that with proper precautions, it is safe and pragmatic to keep a certain level of elective medical services during pandemic. This could also serve as a reference on how to resume OMFS service in a controlled manner when the outbreak is winding down.

As a single-center study, there are intrinsic limitations. The number of trauma cases in our center and the whole Hong Kong is limited, which made the statistical analysis for traumatology as an independent category difficult. There was no COVID-19-positive patient in our cohort. If a multicenter study could be conducted in the future, more comprehensive and widely applicable experience in OMFS patient management in COVID-19 era will be more valuable. Our study only investigated hospital admissions and operations. When the outbreak period is prolonged, data on outpatient management will be needed to guide the outpatient services, which could be time-dependent and lifesaving.

Compared with that pre-COVID-19, the number of admissions and surgeries decreased in the COVID-19 era. There was a significant difference in the disease pattern of OMFS with an increase in the proportion of malignancy cases and essential services. Our results also support that with proper precautions, non-essential medical services can be maintained during the pandemic. Our study has implication for policy and practice for OMFS with impact on patient care, education, and training.

## Data Availability Statement

The raw data supporting the conclusions of this article will be made available by the authors, without undue reservation.

## Ethics Statement

The studies involving human participants were reviewed and approved by Institutional Review Board of the University of Hong Kong (IRB Reference Number: UW 20-684), with the patient informed consent waived. Written informed consent from the participants' legal guardian/next of kin was not required to participate in this study in accordance with the national legislation and the institutional requirements.

## Author Contributions

JJP: conceptualization, methodology, data curation, formal analysis, writing—original draft, and writing—review and editing. CPM: conceptualization, formal analysis, project administration, validation, and writing—review and editing. YYL, WSC, and WFY: project administration, supervision, validation, and writing—review and editing. YXS: conceptualization, formal analysis, funding acquisition, supervision, validation, and writing—review and editing. KYL: statistical analysis and validation. All authors approved the final manuscript for publication.

## Conflict of Interest

The authors declare that the research was conducted in the absence of any commercial or financial relationships that could be construed as a potential conflict of interest.
